# c-Mpl and TPO expression in the human central nervous system neurons inhibits neuronal apoptosis

**DOI:** 10.18632/aging.103086

**Published:** 2020-04-27

**Authors:** Liang Li, Chenju Yi, WenJie Xia, Bihui Huang, Shichao Chen, Junyan Zhong, Xiaoyi Fang, Liuming Yang, Hongwu Xin, Shiying Silvia Zheng, Beng H Chong, Yingyun Fu, Chun Chen, Mo Yang

**Affiliations:** 1 The Seventh Affiliated Hospital, Sun Yat-sen University, Shenzhen, Guangdong, China; 2 Guangzhou Blood Center, Guangzhou, Guangdong, China; 3 Lianjiang People’s Hospital, Lianjiang, Guangdong, China; 4 St. George and Sutherland Clinical School, University of New South Wales, Kogarah, NSW, Australia; 5 Department of Haematology, St. George Hospital, Kogarah, NSW, Australia; 6 Department of Pulmonary and Critical Care Medicine, Shenzhen People’s Hospital, Shenzhen, Guangdong, China

**Keywords:** thrombopoietin, C-Mpl, CNS, brain damage, antiapoptosis

## Abstract

Thrombopoietin (TPO) is a growth factor for the megakaryocytic/platelet lineage. In this study, we investigated the expression of TPO and its receptor, c-Mpl, in the human central nervous system (CNS) and their roles after a neural insult. Our results demonstrate that both TPO and c-Mpl are expressed in the neurons of the human CNS. TPO was also detected in human cerebrospinal fluid. TPO was found to be neuroprotective in hypoxic-ischemic neonatal rat brain models. In these rat models, treatment with TPO reduced brain damage and improved sensorimotor functions. In addition, TPO promoted C17.2 cell proliferation through activation of the PI3K/Akt signaling pathway. Via the Bcl-2/BAX signaling pathway, TPO exerted an antiapoptotic effect by suppressing mitochondrial membrane potentials. Taken together, our results indicate that TPO is neuroprotective in the CNS.

## INTRODUCTION

Thrombopoietin (TPO) is a primary regulator of megakaryopoiesis and thrombopoiesis and is a ligand for the receptor c-Mpl [1, 2]. TPO was first purified in 1994, and since then, much has been learned about its structure, functions, and clinical uses [3, 4]. TPO promotes megakaryocyte lineage differentiation [5] and platelet production [6, 7]. TPO is also essential for bone marrow hematopoietic stem cell (HSC) maintenance. When TPO was deleted from hepatocytes in TPO^DsRed-CreER^ knock-in mice, bone marrow HSCs were depleted [8]. In addition, TPO protects endothelial cells from apoptosis [9, 10]. We have also reported that TPO has an antiapoptotic effect in cardiomyocytes [11].

However, the mechanism by which TPO is protective is not well understood. In a previous study, we demonstrated that megakaryocytes and neurons possess common antigens, such as MAP2, GFAP, Tau, 5-HT2A, 5-HT2B, and 5-HT2C receptors, as well as dopamine D1 and D2 receptors [12–14]. Notably, the TPO receptor c-Mpl is also expressed in the central nervous system (CNS) [15, 16]. Thus, TPO may protect the murine CNS.

In this study, we demonstrate that c-Mpl is expressed in human CNS neurons. We also show that TPO protects the neonatal brain by suppressing apoptosis and that this antiapoptotic effect is mediated through the Bcl-2/BAX axis.

## RESULTS

### Expression of TPO and c-Mpl in human CNS

**Figure 1:**
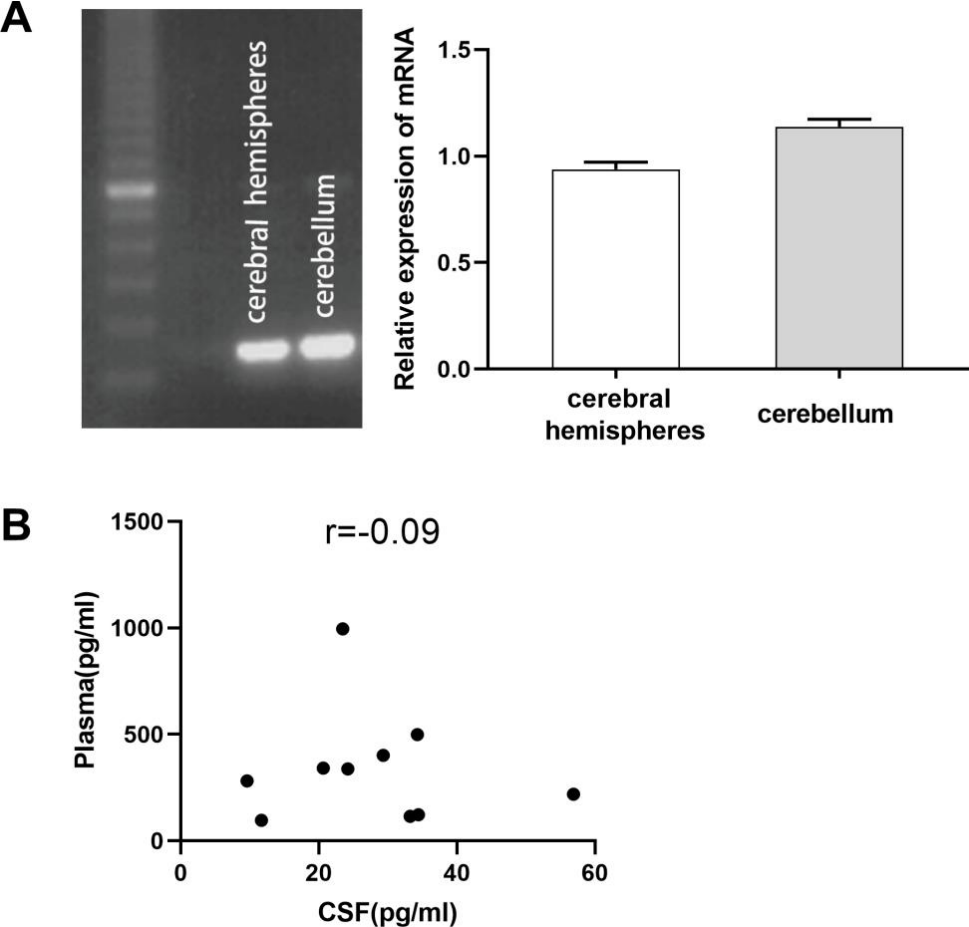
**Expression of thrombopoietin (TPO) mRNA in human cerebral hemisphere and cerebellum.** There was no correlation between the TPO levels in blood plasma and cerebrospinal fluid (CSF). (**A**) TPO mRNA expression in human cerebral hemisphere and cerebellum was detected by RT-PCR, n = 3. (**B**) Scatter plot of TPO levels in blood plasma and CSF, r = –0.09, *P* = 0.80, n = 10.

To investigate the effect of TPO on CNS damage, we first determined its expression in the human CNS. TPO mRNA was detected in the human cerebral hemisphere and cerebellum (Figure 1A). TPO mRNA expression in the cerebellum was found to be higher than that in the cerebral hemisphere. Furthermore, TPO protein was also detected in human cerebrospinal fluid (CSF) (n = 10) and blood plasma (n = 10) (Table 1). The TPO level in CSF (27.75 ± 4.27 pg/mL) was significantly lower than that in blood plasma (341.33 ± 83.86 pg/mL). However, there was no correlation between the TPO levels in CSF and blood plasma in the same patient (Figure 1B, r = –0.09).

**Table 1:** TPO levels in human CSF (n = 10) and plasma (n = 10) by ELISA.

**0BCases**	**CSF (pg/mL)**	**Plasma (pg/mL)**
1	9.58	281.36
2	20.63	342.28
3	29.32	401.37
4	23.44	995.54
5	56.91	218.83
6	34.42	123.51
7	34.26	499.45
8	24.19	337.62
9	11.68	97.44
10	33.21	115.93
Mean ± SEM	27.75 ± 4.27	341.33 ± 83.86

**Figure 2:**
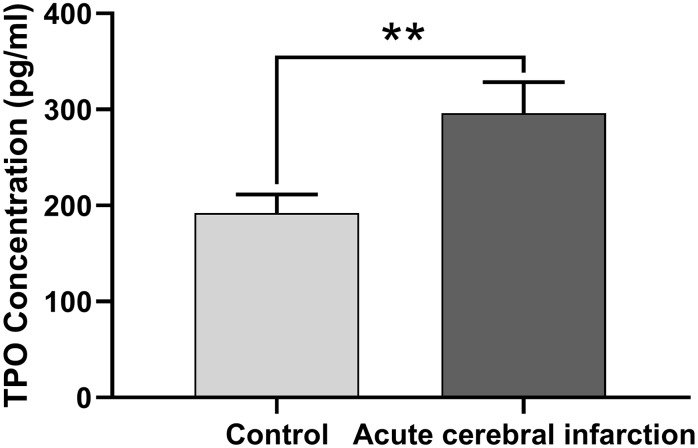
**TPO levels in patients with acute cerebral infarction were higher than those in normal people.** TPO levels in patients (n = 16) and normal people (control group, n = 45) were detected by ELISA. ** *P* < 0.01.

Next, we determined whether TPO levels were different in patients with acute cerebral infarction compared with controls. Patients with acute cerebral infarction (n = 16) had significantly higher levels of serum TPO (296.22 ± 32.32 pg/mL) compared to the control group (n = 45; 192.26 ± 19.40 pg/mL, *P* < 0.01, Figure 2); however, there were no significant changes in blood cell count (Table 2).

**Table 2:** TPO levels and blood cell count in patients with acute cerebral infarction.

**Group**	**TPO (pg/mL)**	**WBC (×10^9^/L)**	**PLT (×10^9^/L)**	**RBC (×10^12^/L)**
Acute Cerebral Infarction (n=16)	296.22 ± 32.32	7.53 ± 1.39	220.94 ± 26.48	4.64 ± 0.31
Control (n=45)	192.26 ± 19.40	7.35 ± 1.49	217.38 ± 32.89	4.52 ± 0.37

**Figure 3:**
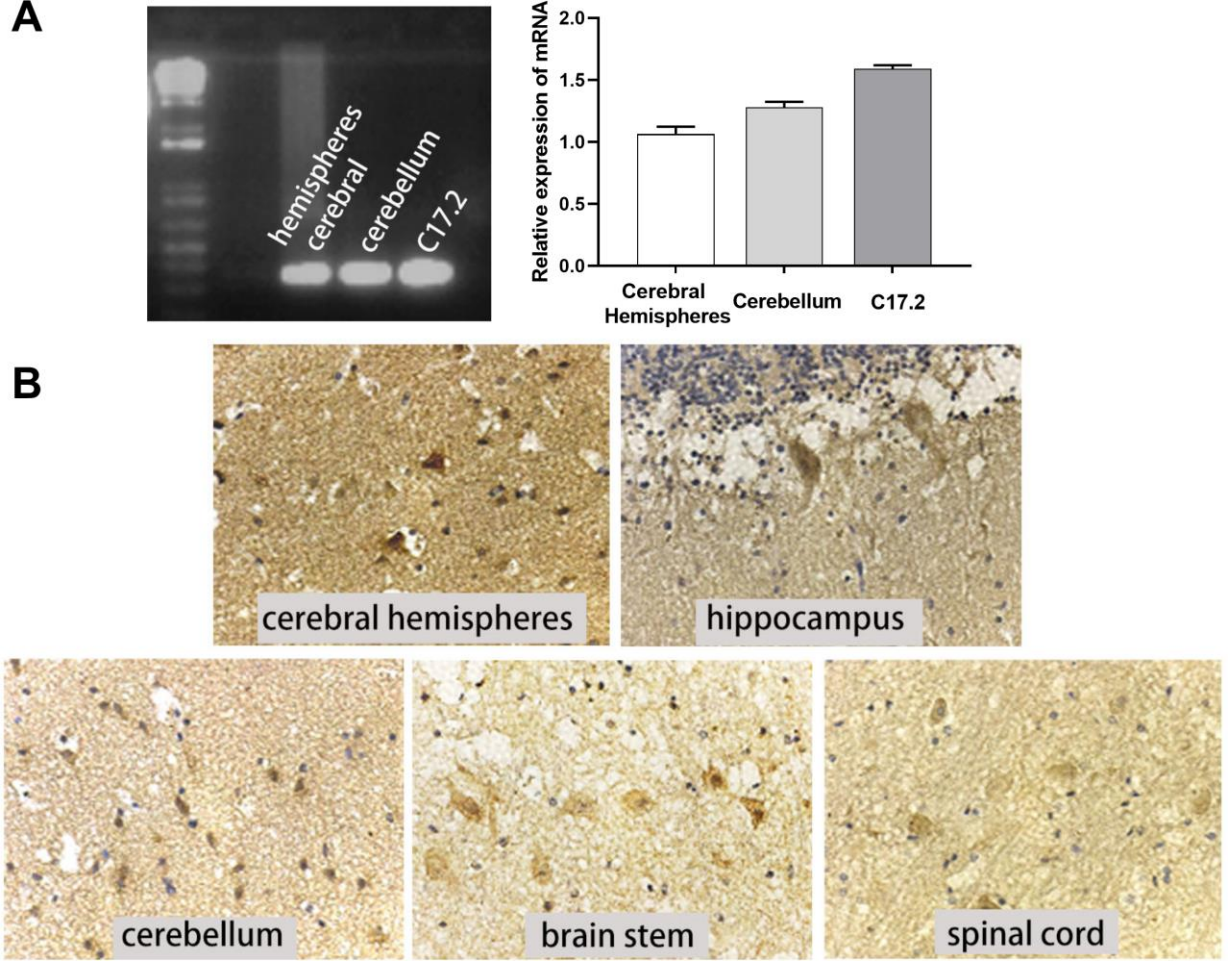
**c-Mpl mRNA and protein were expressed in neural cells and tissues.** (**A**) c-Mpl mRNA expression in human cerebral hemisphere, cerebellum, and C17.2 cells was detected by RT-PCR, n = 3. (**B**) c-Mpl protein expression in human cerebral hemispheres, hippocampus, cerebellum, brain stem, and spinal cord was detected by immunohistochemistry.

TPO, thrombopoietin; WBC, white blood cell; PLT, platelet; RBC, red blood cell.

c-Mpl is a major receptor that mediates the response to TPO; thus, we also measured c-Mpl expression in human CNS tissues and cell lines. c-Mpl mRNA expression was found in human cerebral hemispheres, cerebellum, and C17.2 cells (Figure 3A). More importantly, we detected c-Mpl protein expression in neurons in human cerebral hemispheres, hippocampus, cerebellum, brain stem, and spinal cord (Figure 3B). Hippocampal neurons had the highest levels of c-Mpl protein.

### Effect of TPO in neonatal hypoxic-ischemic rat model

After we confirmed TPO and c-Mpl expression in the human CNS and found that TPO expression was increased in patients with acute cerebral infarction, to further investigate TPO’s effect in pathologies, we established a neonatal rat model of hypoxic-ischemic brain damage. The mortality rates of rats in the vehicle-treated and TPO-treated groups were 12.0% and 11.0%, respectively (n = 16). These rats died either during surgery or from hypoxia. Among the surviving rats in the treatment and sham-operated control groups, no difference in total body weight was seen, with a mean range of 23.3-24.4 g at 1 week and 96.4-100 g at 3 weeks after surgery. No discernable physiologic or behavioral changes due to toxication were observed. These results indicate that a successful model was established.

**Figure 4:**
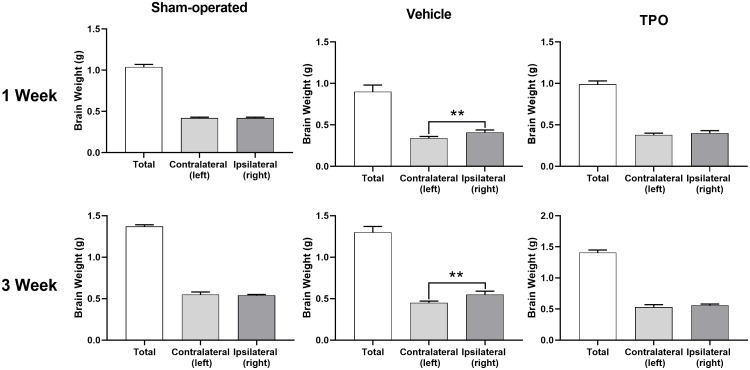
**TPO demonstrated a neuroprotective effect in a neonatal hypoxic-ischemic rat model.** Brain injury was estimated using the percentage of the weight reduction in the ipsilateral cerebral hemisphere compared to the contralateral hemisphere. Brain weights of ipsilateral cerebral hemisphere and contralateral hemisphere were measured in the sham-operated, vehicle-treated, and TPO-treated rats at 1 and 3 weeks of hypoxia-ischemia, n = 16. ** *P* < 0.01.

Brain injury was estimated using the percentage of weight reduction in the ipsilateral cerebral hemisphere compared to the contralateral hemisphere. At both assessment time points (1 and 3 weeks after hypoxic-ischemic treatment), the weights of the ipsilateral hemisphere (hypoxic-ischemia side) of the vehicle group decreased significantly compared with those in the sham group (Figure 4). Pups treated with TPO for 9 or 16 days had significantly higher weights in the ipsilateral hemisphere compared with those in the vehicle group (*P* < 0.05). Similar effects were observed in total brain weight at 3 weeks after surgery (*P* < 0.05). The contralateral brain weights of all groups were similar at both time points. The neuroprotective effect of TPO was consistent at these two time points when brain damage was determined by the reduced weight of the ipsilateral hemisphere compared with the contralateral hemisphere (*P* < 0.01, TPO vs vehicle group).

**Figure 5:**
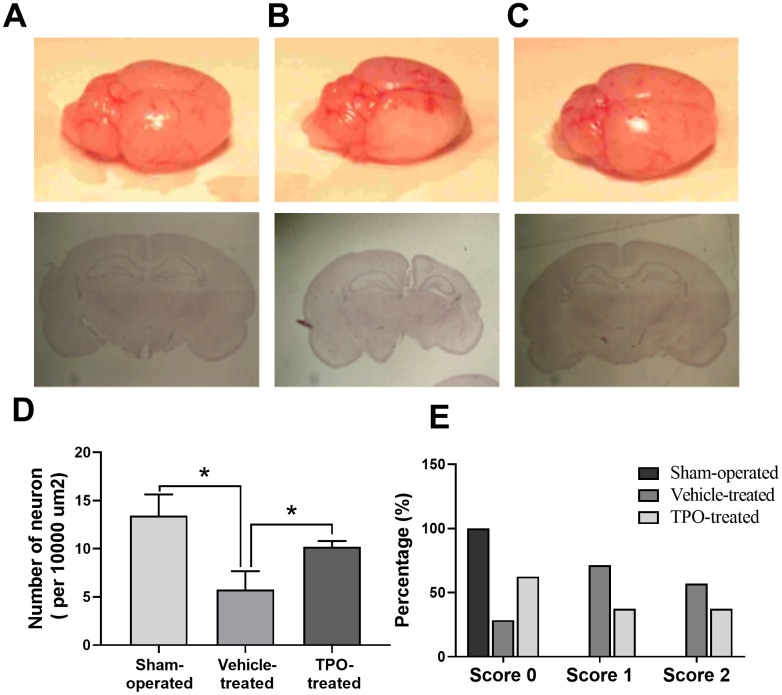
**Brain morphology, histology, NSE staining of cortical neurons, and postural reflex test in sham-operated, vehicle-treated, and TPO-treated pups 3 weeks after surgery.** Brain morphology and histology were examined at 3 weeks after surgery. Cortical neurons in the ipsilateral hemispheres were detected using NSE staining. (**A**) Normal external brain morphology and histology of sham-operated pups. (**B**) Brain morphology and histology of vehicle-treated pups. (**C**) Brain morphology and histology of TPO-treated pups. (**D**) The numbers of cortical neurons in the ipsilateral hemispheres of sham-operated, vehicle-treated, and TPO-treated pups, n = 3. (**E**) Postural reflex test scores in sham-operated (n = 12), vehicle-treated (n = 16), and TPO-treated pups (n = 16). * *P* < 0.05.

Next, we examined brain morphology at 3 weeks after surgery. Vehicle-treated animals displayed severe atrophy in the ipsilateral hemisphere (Figure 5B). In contrast, TPO treatment reversed the ipsilateral atrophy, as determined by the morphology of the right hemisphere, which was similar to that of the sham-operated control (Figure 5C). Neuron-specific enolase (NSE) staining of coronal sections also showed that vehicle-treated hypoxic-ischemic pups suffered from gross deformation and severe neuronal loss in the ipsilateral hemisphere, especially in the outer layer of the cerebral cortex (Figure 5A, 5B, 5D). In contrast, treatment with TPO decreased brain injury and improved neuronal damage (Figure 5C, 5D). The hemispheric structure of the TPO-treated group remained intact despite a slight reduction in size compared to the sham-operated pups (Figure 5C).

Next we examined whether TPO treatment resulted in recovery of brain function and mobility in the hypoxic-ischemic pups by performing the postural reflex test. Vehicle-treated pups exhibited abnormal postural reflex response; 16 of these pups had scores of 1 and 2 (*P* < 0.05), whereas all of the sham-operated pups had scores of 0 (n = 12) (Figure 5E). TPO treatment improved postural reflex, as demonstrated by the increased proportion of pups with a score of 0 and the reduced proportion with scores of 1 or 2 (n = 16, *P* < 0.05). However, TPO treatment did not result in full recovery of brain function to the level of that in sham-operated pups.

### Effect of TPO on neural cells in vitro

**Figure 6:**
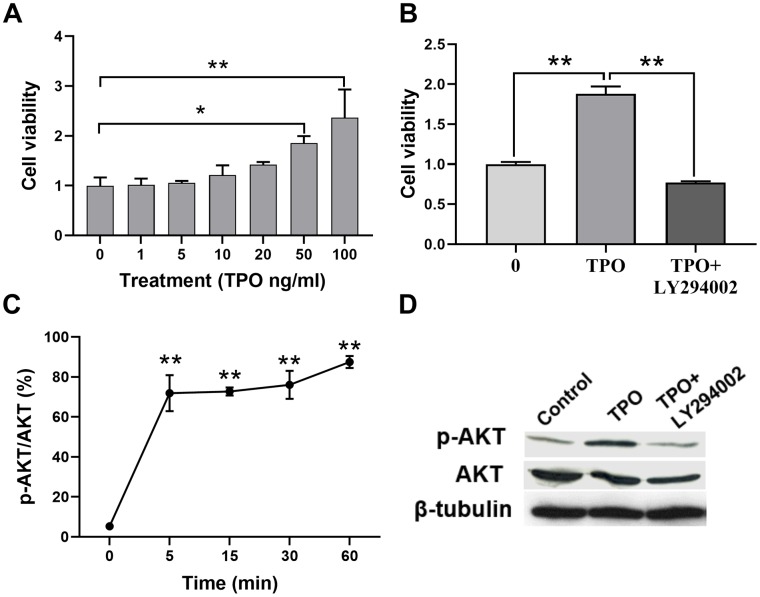
**TPO promoted cell proliferation and activated the PI3K/AKT signal in C17.2 cells.** C17.2 cells were treated with TPO for 72 h. These data were expressed as means ± SEM. Cell viability was detected by MTT. (**A**) Cell viability of C17.2 cells with different TPO concentrations, n = 3. (**B**) Cell viability of C17.2 cells that were treated with the PI3K inhibitor (LY294002, 50 μM) prior to TPO treatment, n = 8. (**C**) Levels of phosphorylated AKT (p-AKT) at different time intervals. (**D**) Levels of AKT and p-AKT in the groups treated with TPO and TPO+LY294002. * *P* < 0.05, ** *P* < 0.01.

We demonstrated that TPO treatment decreases neuronal death and facilitates brain function recovery in a neonatal hypoxic-ischemic rat model. To determine the cellular mechanisms of TPO, we conducted experiments with the C17.2 cell line, an immortalized mouse neural progenitor cell line, because we previously determined that C17.2 cells express the TPO receptor c-Mpl (Figure 3A). TPO has a dose-dependent effect on the growth of C17.2 cells, as demonstrated by an MTT assay (Figure 6A). Previous studies have shown that TPO promotes cell survival through the AKT pathway [17–19]. In line with these observations, we found activation of the AKT pathway in TPO-treated cells (Figure 6C). To confirm that TPO promotes cell survival via the AKT pathway, we pretreated the cells with the PI3K inhibitor LY-294002 before TPO addition. The results showed that LY-294002 pretreatment suppressed the TPO-induced AKT activation and abolished the prosurvival effect of TPO (Figure 6B). These results confirm that TPO promotes cell survival via the PI3K-AKT pathway.

**Figure 7:**
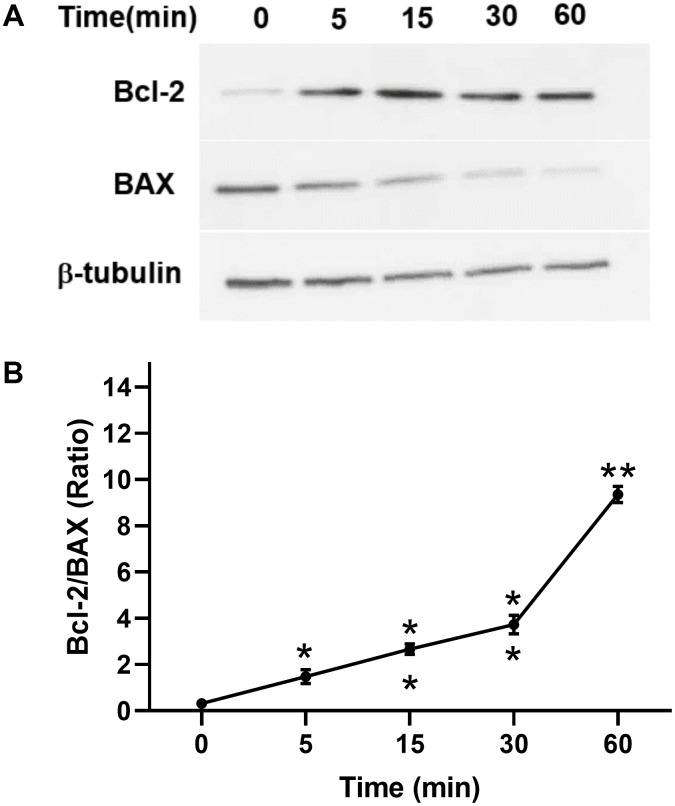
**Effect of TPO on Bcl-2 and BAX of C17.2 cells at different time intervals.** Cells were stimulated for the selected times with 100 ng/mL of TPO. Bcl-2 and BAX were detected by Western blot. (**A**) Levels of Bcl-2 and BAX at different time intervals. (**B**) Semiquantitative evaluation of TPO for antiapoptotic effects via Bcl-2/BAX. * *P* < 0.05, ** *P* < 0.01.

In addition, we investigated the antiapoptotic effect of TPO on C17.2 cells. Annexin V assays showed that TPO treatment (50 ng/mL) reduced the percentage of cell deaths (annexin V+/PI+) and apoptotic cells (annexin V+/PI–) (*P* < 0.05) in serum-free culture conditions (Table 3). These results indicate that TPO promotes survival of C17.2 cells. TPO treatment consistently led to increased levels of Bcl-2 and decreased levels of BAX in a time-dependent manner (0, 5, 15, 30, and 60 mins) in C17.2 cells cultured in serum-free media (Figure 7A, 7B), which indicates that the antiapoptotic effects of TPO on neural cells may be related to Bcl-2/BAX.

**Table 3:** Antiapoptotic effect of TPO on C17.2 cells (n = 5; mean ± SEM).

**Group**	**Control**	**TPO (100 ng/mL)**
Total cell death (annexin V plus PI)	35.2 ± 8.8%	18.1 ± 6.2%
Early cell death (annexin V)	17.0 ± 7.4%*	8.2 ± 5.7%*

*Control group versus TPO-treated group by Student’s t-test, *P* < 0.05.

**Figure 8:**
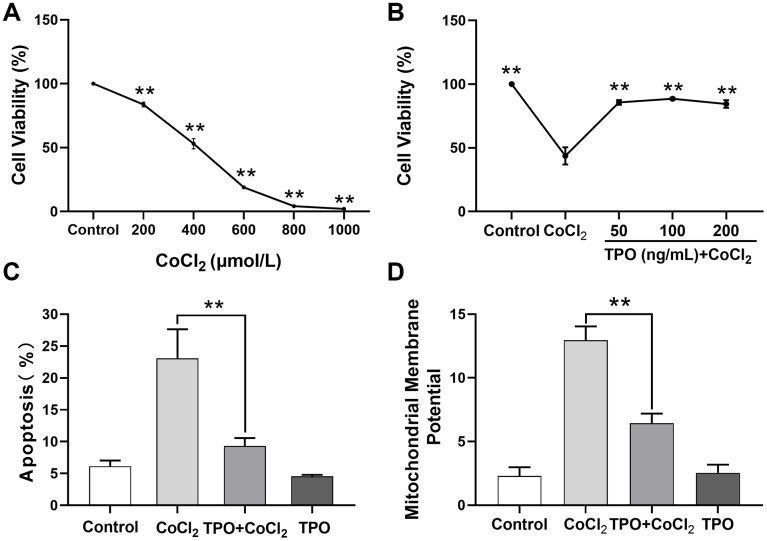
**TPO demonstrated a protective effect in a CoCl_2_-induced PC12 cell injury protection model.** Cell viability was detected by MTT. Cell apoptosis and mitochondrial membrane potential were detected by flow cytometry. (**A**) Effect of different concentrations of CoCl_2_ on viability of PC12 cells. Cells were treated with CoCl_2_ for 24 h, n = 3. (**B**) Protective effect of different concentrations of TPO on CoCl_2_-induced (500 μmol/L) PC12 cells. Cells were treated with TPO for 48 h, n = 3. (**C**) Protective effect of 100 ng/mL of TPO on CoCl_2_-induced (500 μmol/L) PC12 cells. (**D**) Protective effect of 100 ng/mL of TPO on mitochondrial membrane potential of CoCl_2_-induced (500 μmol/L) PC12 cells. ** *P* < 0.01.

Next, we determined the ability of TPO to protect against CoCl_2_-induced cell injury in PC12 cell lines. The 24-hour survival rate of PC12 cells decreased significantly in a dose-dependent manner (Figure 8A).

Based on this result, 500 μmol/L of CoCl_2_ was chosen as the appropriate concentration to establish the chemical hypoxia model in subsequent experiments. A cell viability test showed that the protective effect of TPO on chemical hypoxia in PC12 cells was dose independent at the range of 50 to 200 ng/mL (Figure 8B). In addition, an apoptosis assay showed that TPO treatment significantly reduced the proportion of CoCl_2_-induced apoptotic cells. However, TPO treatment did not significantly reduce the number of apoptotic cells in the control PC12 cells (Figure 8C). In addition, CoCl_2_-induced hypoxia significantly increased the mitochondrial membrane potentials of PC12 cells, but TPO treatment reversed this effect (Figure 8D). Thus, TPO might protect mitochondrial function during hypoxic-ischemic conditions.

## DISCUSSION

In clinical studies, we have found that plasma TPO levels in patients with acute cerebral infarction are significantly increased, indicating that TPO may play an important role in CNS injury. To validate this hypothesis, we first sought to determine the basal level of c-Mpl and TPO in neurons of the CNS of humans and mice. To investigate the effects of TPO in the CNS, we established a neonatal rat model of hypoxic-ischemic brain injury induced at postnatal day 7 [20]. In our study, rat pups treated with TPO before the induction of hypoxia-ischemia for 9 days (2 days prior to surgery plus 1 week) or 23 days (2 days prior to surgery plus 3 weeks) demonstrated a remarkable recovery of the ipsilateral hemispheres in all studied parameters. TPO treatment reduced atrophy of the right hemisphere, which was almost returned to its normal size and morphology. In addition, treatment with TPO significantly increased the number of neurons. More significantly, animals treated with TPO performed better on functional testing when compared to the vehicle-treated group. These data suggest that TPO provides histopathologic and behavioral protection against neonatal hypoxic-ischemic brain injury in vivo.

Our findings raise the question of how TPO protects the brain from hypoxic-ischemic injury. To answer this question, we performed experiments in two neural cell lines: C17.2 and PC12 cells [21, 22]. TPO has been shown to inhibit apoptosis in these two cell lines. Our results demonstrate that TPO activates AKT signaling, which may represent a critical step in suppressing apoptosis, and improves the ratio of Bcl-2/BAX on the apoptotic axis. TPO inhibited apoptosis of C17.2 cells by increasing antiapoptotic Bcl-2 expression and decreasing proapoptotic BAX expression [23, 24]. The PI3K-AKT signal transduction pathway is also important for promoting cell survival [25]. Activation of AKT results in the inhibition of several cellular factors that lead to the suppression of apoptosis [26]. Moreover, TPO protects PC12 cells from hypoxic damage by inhibiting the increase of mitochondrial membrane potentials. Changes in mitochondrial membrane potentials affect apoptosis [27]. Mitochondrial dysfunction has been shown to induce apoptosis and has even been suggested to be central in the apoptotic pathway [28]. Taken together, these results suggest that TPO protects neural cells by activating PI3K-AKT and Bcl-2-BAX.

The genomic structures of TPO and erythropoietin (EPO) are highly similar at most segments of their coding regions and exhibit significant homology in their receptor binding domains [29, 30]. Both the EPO receptor and the TPO receptor come from the same cytokine receptor superfamily [31, 32]. EPO is the main hematopoietic cytokine that regulates the formation of red blood cells in the process of hematopoiesis [33]. EPO activates red blood progenitor cells by stimulating cell growth, differentiation, and antiapoptosis [34]. Recent findings indicate that EPO is neuroprotective and facilitates brain repair [35, 36]. EPO protects neurons from glutamate toxicity in vitro and has neurotrophic effects in global and focal cerebral ischemia [37]. Thus, TPO may have similar neuroprotective mechanisms as EPO.

Other studies have indicated that TPO is neuroprotective during brain injury. In a rat model of severe infarction and swelling after stroke induced by middle cerebral artery occlusion reperfusion, TPO significantly reduced both the infarct and the swelling in a dose-dependent manner [38]. When 0.1 μg/kg of TPO was administrated immediately or 2 hours after reperfusion, the infarct and swelling were significantly improved, and other stroke-related neurologic deficits were also ameliorated [38, 39]. Treatment with TPO also reduces stroke-induced cortical MMP-9 and TIMP-1 expression and enzymatic activity. Our study provides corroborative evidence that TPO protects neural cells from apoptosis and reduces brain damage in neonatal models.

The neuroprotective mechanism of TPO requires further investigation to fully understand its role in the CNS. However, studies in other systems may help to shed light on the underlying mechanism. Our previous data from a rat model demonstrated that TPO reduces damage to heart tissues caused by doxorubicin-induced cardiotoxicity and reduces myocardial infarction damage [11, 40]. TPO also protects against iron overload–induced apoptosis by inhibiting oxidative stress and suppressing mitochondrial pathways in cardiomyocytes [41] and protects H9C2 cells from excessive autophagy and apoptosis in doxorubicin-induced cardiotoxicity [11, 42]. TPO also confers immediate protection to human cardiomyocytes against injury from hypoxia/reoxygenation by decreasing necrotic and apoptotic cell death in a concentration-dependent manner, with an optimal concentration of 1.0 ng/mL [43]. In addition, TPO activates endothelial cells and induces an angiogenic response by enhancing expression of vascular endothelial growth factor (VEGF) in primitive hematopoietic cells through induction of HIF-1 [10, 44].

This study demonstrates that TPO protects neural cells from apoptosis, further elucidates the role of TPO in the CNS, and provides insight on possible clinical uses of TPO.

## MATERIALS AND METHODS

### Cell lines

The mouse neural progenitor cell line C17.2 was a gift from Dr. David Walsh (Department of Anatomy, University of New South Wales, Sydney, Australia). PC12 cells were obtained from the Sun Yet-Sen University School of Medicine. C17.2 cells were cultured in Iscove’s Modified Dulbecco’s Medium (IMDM) (Gibco; Thermo Fisher Scientific, Waltham, MA) supplemented with 10% (v/v) fetal calf serum (FCS) (Gibco), and PC12 cells were cultured in Roswell Park Memorial Institute (RPMI) 1640 Medium with 10% (v/v) fetal bovine serum (FBS) (Gibco) in an atmosphere of 5% CO_2_/95% humidified air at 37°C.

### Human brain tissues and cerebrospinal fluid

Human brain tissues were obtained from the Department of Anatomical and Cellular Pathology at the Prince of Wales Hospital, associated with the Chinese University of Hong Kong. Human cerebrospinal fluid (CSF) and plasma were obtained from children with acute lymphocytic leukemia in the Department of Pediatrics at the Prince of Wales Hospital. Patients with acute cerebral infarction were treated as a case group, and those with healthy physical examination at the same time were treated as a control group (Luohu People’s Hospital, Shenzhen). Informed consent was obtained from the patients and their families for all blood and tissue collections, and the study was approved by the Ethics Committee for Clinical Research of Sun Yat-sen University.

### RT-PCR for TPO and c-Mpl mRNA expression

Total cellular RNA was extracted, and reverse transcriptase polymerase chain reaction (RT-PCR) was performed as previously described [45]. The RNA pellet was resuspended in 50 μL of DEPC-treated water. The first-strand cDNA was synthesized from total cellular RNA using SuperScript II reverse transcriptase (Gibco). RT-PCR was performed using primers specific for the TPO and c-Mpl sequence: TPO forward primer 5′-CTGCTTCGTGACTCCCATGTC-3′ and reverse primer 5′-CGCACCTTTCCTCGGAGCAG-3′, c-Mpl forward primer 5′-CTAGCTCCCAAGGCTTCTTC-3′ and reverse primer 5′-GGCTCCAGCACCTTCCAGTCC-3′.

### Immunohistochemistry for c-Mpl on human CNS

The human brain was removed from the skull, placed in 10% neutral formaldehyde overnight at room temperature, and processed for paraffin histology. The brain was cut into 5-μm sections and deparaffinized in xylene and graded alcohol before immersion in citrate buffer (pH 7.6) for antigen retrieval in a microwave oven. The sections were placed in 3% hydrogen peroxide for 20 min to block endogenous peroxidase and then incubated in 5% rabbit serum (Dako, Glostrup, Demark) for 10 min. Sections were stained with primary fluorescent monoclonal antibody against c-Mpl (1:100 dilution; BD Pharmingen, San Diego, CA) and imaged by confocal microscopy. The nonfluorescent primary monoclonal antibody against c-Mpl (1:200 dilution; BD Pharmingen) was added onto the sections, which were then incubated overnight at room temperature. The sections were further treated with a biotinylated rabbit antimouse antibody (1:1000 dilution; Dako, Glostrup, Demark) for 40 min before incubation with horseradish peroxidase (HRP; Zymed, San Francisco, CA) for 45 min. Color development was performed in 3,3′-diaminobenzidine tetrahydrochloride (Sigma-Aldrich, St. Louis, MO) solution for 10 min. After staining, the sections were washed, and coverslips were applied with Permount (Fisher Scientific, Loughborough, UK).

ELISA assay for TPO: TPO levels were measured with an enzyme-linked immunosorbent assay (ELISA) kit (R&D, Minneapolis, MN). The assay was done as per manufacturer’s instructions. A monoclonal antibody specific for TPO was precoated onto a microplate. Standards and samples were pipetted into the wells, and any TPO present was bound by the immobilized antibody. After washing away unbound substances, an enzyme-linked polyclonal antibody specific for TPO was added to the wells. After a wash to remove any unbound antibody-enzyme reagent, a substrate solution was added to the wells and color developed in proportion to the amount of TPO bound in the well. The optical density of each well was measured at 450 nm by a microreader (BioTek Instruments, Winooski, VT).

### MTT assay

C17.2 cells were seeded onto 96-well culture plates at 5000 cells per well. After being cultured in IMDM with 10% FCS overnight, the cells were washed twice with PBS and then incubated without serum [21, 46]. Escalating doses of TPO (Pepro Tech, Rocky Hill, NJ; 0, 1, 10, 50, 100, and 200 ng/mL) were added to each well and incubated for 72 hours. C17.2 cells were pretreated with 50 μM of LY294002 (Calbiochem, Darmstadt, Germany), followed by 100 ng/mL of TPO, and then incubated for 72 hours. For the MTT proliferative activity assay, cells were incubated with 3-(4,5-dimethylthiazol-2-yl)-2,5-diphenyl tetrazolium bromide (MTT; Sigma-Aldrich) for 4 h. After 4 h, the supernatant was discarded, and DMSO (200 μL) was added to each well. The suspension was placed on a microvibrator for 5 min, and the absorbance (A) was measured at 570 nm with a microplate spectrophotometer (μ-Quant Microplate Spectrophotometer; BioTek Instruments). Cell viability was calculated using the following formula:

% Cell viability = Experimental MTT (OD570)/Normal MTT (OD570)

### Assessment of cell survival and apoptosis

A total of 1.0 × 10^6^ C17.2 cells treated with TPO (100 ng/mL), as described earlier, were examined for apoptosis using an annexin V-FITC detection kit (BD Biosciences, San Diego, CA) according to manufacturer’s instructions. Briefly, cells were counted and resuspended in 500 μL of cell culture medium, and annexin V-FITC plus a binding enhancer were added directly to the cells for 20 min. Propidium iodide (PI) was added as described earlier, and the cells were analyzed with a FACScan cytometer (BD Immunocytometry Systems, San Jose, CA) [45]. The cells were kept on ice (approximately 30-60 min) until the FACScan analysis was completed.

### Western blot

For AKT, p-AKT, Bcl-2, and BAX immunodetection, cells were plated at initial densities of 5.0 × 10^5^ cells in 35-mm diameter plates and serum-starved overnight. When needed, a 30-min preincubation step with the PI3K inhibitor LY294002 was included before stimulation. Cells were stimulated for the selected times with the indicated TPO treatment of 100 ng/mL. Then, they were rinsed rapidly in ice-cold PBS and lysed in a buffer containing 2% sodium dodecyl sulfate (SDS; Sigma-Aldrich) and 125 mM Tris (pH 6.8) buffer. Lysates were sonicated, and protein was quantified using the DC Protein Assay from Bio-Rad (Hercules, CA). Cell lysates were resolved by SDS-polyacrylamide gel electrophoresis. Membranes were blocked with Tris-buffered saline with Tween 20, 20 mM Tris–HCl (pH 7.4), 150 mM NaCl, and 0.05% Tween 20 containing 5% nonfat dry milk for 1 h at room temperature. Membranes were probed with the appropriate primary antibodies (1:1000; Santa Cruz Biotechnology, Dallas, TX) overnight and subsequently incubated for 1 h with the appropriate peroxidase-conjugated secondary antibodies (1:1000) at the dilutions recommended by the manufacturers. Blots were finally developed with an ECL (Amersham Biosciences, Little Chalfont, UK) Western blotting detection system [45].

### Animal protocols

All procedures were carried out in accordance with guidelines approved by the Animal Ethics Committee of the Chinese University of Hong Kong. Sprague-Dawley rat pups were kept with their dams in the Laboratory Animal Service Center with a light:dark cycle of 12:12 h and allowed food and water *ad libitum*.

### Induction of hypoxia-ischemia in neonatal rats

Hypoxic-ischemic brain damage was induced in rat pups (weighing 12-15 g) on postnatal day 7 [47, 48]. At this stage of development, the rat brain is histologically similar to that of a 32- to 34-week gestation human infant. The rat pups were anesthetized using ether. The right common carotid artery was exposed and ligated with size 4-0 surgical sutures. The entire procedure was completed in less than 10 min. After carotid ligation, the pups were returned to their dams and allowed to recover for 2 h. Hypoxia was then induced by exposing the animals to a humidified gas mixture containing 8% oxygen in nitrogen at 37°C for 2 h. The pups were returned to their dams after hypoxic exposure. Sham-operated pups underwent the same surgical procedure but did not receive carotid ligation or exposure to hypoxia.

### Administration of TPO

The rat pups were randomly allocated to one of three groups: sham-operated group (n = 12), vehicle-treated group (PBS; n = 16), or TPO-treated group (n = 16). TPO (Pepro Tech) was administered daily by intraperitoneal injection at a dose of 1 μg/kg/d beginning on postnatal day 5 (2 days prior to surgery) for 9 or 23 days, and the animals were killed at 1 or 3 weeks after surgery with ketamine (0.05 mL/kg) and xylazine (0.01 mg/kg) (Alfasan, Woerden, the Netherlands).

### Brain weight

The cerebral hemispheres, brain stem, and cerebellum were removed from the skull. The hemispheres were separated by a longitudinal midline incision, and each hemisphere was weighed on a high-precision digital balance (sensitivity ± 0.001 g). The difference in weights between the ipsilateral (right) and contralateral (left) brain was calculated using the following formula:

% Damage = (C – I)/C × 100,

where C and I denote weights of the contralateral and ipsilateral hemispheres, respectively.

### Histology

Rat brains at 3 weeks postsurgery were fixed in 10% neutral formaldehyde and kept at 4°C. Coronal blocks (2 mm thick) were cut from the brain, with the most frontal cut being 2 mm from the frontal pole of the intact hemisphere. The tissue blocks were dehydrated in 70% ethanol, embedded in paraffin wax, and sectioned into 5-μm slices. Sections were stained with hematoxylin and eosin and examined under light microscopy [49].

### Counting of cortical neurons in the sensorimotor area of the forelimb

Three weeks after surgery, rats were anesthetized with ketamine (0.05 mL/kg) and xylazine (0.01 mg/kg) and then transcardially infused with 0.9% saline followed by 10% ice-old neutral formaldehyde (Sigma, St. Louis, MO). Their brains were removed, placed in 10% neutral formaldehyde overnight at room temperature, and processed for paraffin histology. Then, 5-μm sections were stained with neuron-specific enolase (NSE) using the method described earlier. The cortical neurons in the sensorimotor area of the forelimb were counted in five randomly selected frontal sections by investigators who were blinded to the allocation of treatment groups. These neurons were identified by their location, size, and NSE staining. The neuron density of each group (three animals) was expressed as the mean number of neurons per 10,000 μm^2^.

### Functional test

A standard postural reflex test was performed to evaluate the extent of neural recovery in rat pups 3 weeks after surgery [50, 51]. The investigator had no prior information on the treatment of the rats. The pup was held by the tail 50 cm above a table. Normal rat pups extended both forelimbs toward the table (score 0). Pups with brain damage flexed the forelimb contralateral to the damaged hemisphere (score 1). The pup was then put onto the table, and a lateral pressure was applied behind the shoulder until the forelimbs slid. A reduced resistance to this lateral force toward the left side (contralateral to the brain damage) was considered abnormal (score 2). Results are presented as the percentage of rats in each of the functional groups.

### Statistical analysis

All values are presented as mean ± SEM. Statistical analysis was performed using a two-tailed unpaired Student’s t-test or analysis of variance (ANOVA) for multiple comparisons. *P* < 0.05 was considered statistically significant.

### Ethics, consent, and permissions

The Animal Research Welfare Committee of Sun Yat-sen University approved this experimental protocol. The principles of the National Institutes of Health Guidelines for Laboratory Animals were followed during the entire course of the experiment. All parts of this report are basically in compliance with the ARRIVE Guidelines for reporting animal research.
